# A systematic review of the social determinants of physical and mental health in women with schizophrenia: focus on housing conditions

**DOI:** 10.1192/j.eurpsy.2025.898

**Published:** 2025-08-26

**Authors:** A. González-Rodríguez, M. Natividad, M. G. Jiménez, L. C. Susser, R. Ayesa-Arriola, E. Izquierdo, A. Balagué, J. P. Paolini San Miguel, N. Bagué, A. Pérez, M. Salvador, J. A. Monreal

**Affiliations:** 1 Mental Health, Mutua Terrassa University Hospital. University of Barcelona. CIBERSAM; 2 Mental Health, Mutua Terrassa University Hospital. University of Barcelona, Terrassa; 3 Faculty of Education, International University of La Rioja, Logroño, Spain; 4 Psychiatry, New York-Presbyterian/Weill Cornell Medical Center. White Plains, New York, United States; 5 Psychiatry, Marqués de Valdecilla University Hospital, IDIVAL, School of Medicine, University of Cantabria., Santander, Spain

## Abstract

**Introduction:**

The ecological hypothesis for schizophrenia supports the relationship between the urban environment and the clinical expression of severe psychosis. Housing conditions have been poorly studied.

**Objectives:**

Our aim was to investigate the impact of housing conditions on schizophrenia, particularly in women.

**Methods:**

A systematic literature search was conducted in PubMed, Scopus and ClinicalTrials.gov from inception to July 2024 according to the PRISMA guidelines. Search terms: (housing conditions) OR (poor housing) AND health AND schizophrenia.

**Results:**

The search yielded 301 articles, from which 16 were included. Only three studies reported results specifically to women.

1) Building (n=4). Poor housing conditions associated with better self-esteem (n=1), but increased incidence of schizophrenia in African-Caribbeans (n=1). Despite difficulties in accessing adequate housing (n=1), schizophrenia patients showed high resilience (n=1).

2) Housing environment (n=4). Living in deprived neighbourhoods associated with higher negative symptoms (n=1) and poor community adjustment (n=2). Importance of the house’s proximity to places for recreation (n=1).

3) Living in group/independent housing (n=3). Women living in institutions need more physical care than men (n=2). Living in shared accommodation reduces social loneliness and quality of life (QoL) (n=1).

4) Private homes/ boarding houses (n=3). Boarding houses are the least preferred type of community accommodation compared to private homes (n=3).

5) Social support/QoL (n=2). Lower QoL is associated with non-institutional housing (n=1). Housing type may influence cognitive function (n=1).

**Conclusions:**

Inadequate housing is negatively associated with mental health outcomes in schizophrenia. Few studies have investigated light, ventilation and internet access with health and QoL. Future studies should investigate housing conditions, especially in women.

**Disclosure of Interest:**

None Declared

